# Optothermal Raman Spectroscopy of Black Phosphorus on a Gold Substrate

**DOI:** 10.3390/nano12091410

**Published:** 2022-04-20

**Authors:** Emiliano Bonera, Alessandro Molle

**Affiliations:** 1 Dipartimento di Scienza dei Materiali and L-NESS, Università degli Studi di Milano-Bicocca, Via R. Cozzi 55, 20125 Milano, Italy; 2 CNR-IMM, Unità di Agrate Brianza, Via C. Olivetti 2, 20864 Agrate Brianza, Italy

**Keywords:** black phosphorus, interface thermal resistance, finite-element method, optothermal raman spectroscopy, phosphorene, Xenes

## Abstract

With black phosphorus being a promising two-dimensional layered semiconductor for application to electronics and optoelectronics, an issue remains as to how heat diffusion is managed when black phosphorus is interfaced with metals, namely in a typical device heterojunction. We use Raman spectroscopy to investigate how the laser-induced heat affects the phonon modes at the interface by comparing the experimental data with a finite element simulation based on a localized heat diffusion. The best convergence is found taking into account an effective interface thermal conductance, thus indicating that heat dissipation at the Au-supported black phosphorus nanosheets is limited by interface effect.

## 1. Introduction

Black phosphorus (BP) is the orthorhombic allotrope of phosphorus. Due to its layered character, it can be reduced to the ultimate form of a two-dimensional (2D) crystal, known as phosphorene [1]. Phosphorene generally belongs to the class of the Xenes, 2D mono-elemental crystals, within the column of pnictogens in the periodic table, and, as such, it holds potential for a wealth of nanotechnology applications beyond graphene [2,3,4,5,6,7,8,9]. Unlike graphene, phosphorene, as derived from the BP layer exfoliation, looks like a puckered lattice endowed with an inherent structural anisotropy [10] and a thickness-dependent energy gap spanning from 0.3 eV in the multilayer (bulk regime) to 1.5 eV in the single layer. The two latter features make BP particularly interesting for applications in electronics and optoelectronics based on 2D materials, either as a counterpart of the semimetallic graphene or as a competitor of other 2D semiconducting compounds, such as transition metal dichalcogenides. Not only do BP nanosheets display photoconductivity [11,12], but also the hole carriers in BP display a high mobility when they flow through as channel carriers in a field-effect transistor structure [13,14].

This opto-electronic performance has been investigated also in conjunction with the thermal properties, demonstrating promising thermoelectric features. In this respect, phosphorene is expected to gain a good trade-off between a relatively high electrical conductivity and a concomitantly low thermal conductivity, thereby maximizing the thermoelectric figure of merit zT factor [15,16], defined as zT = S^2^ s T/*k*, where S, s, T, and *k* are the Seebeck coefficient, the electrical conductivity, the absolute temperature, and the thermal conductivity, respectively. It is reported that the electrical conductance along the armchair direction is from 2 to 10 times greater than that along the zigzag direction, whereas the thermal conductivity along the zigzag direction is higher than that along the armchair direction [17,18]. In addition to thermoelectricity, the thermal conductivity is also related to how BP can efficiently dissipate heat in a device. This aspect is particularly relevant when BP is in contact with other materials, as in the case of the ubiquitous metal electrodes that must be integrated in any kind of electronic and optoelectronic devices where BP may serve as a key component. However, an experimental work investigating the heat dissipation through a stack composed of BP and metal is still missing.

Accessing the thermal properties of a microscopic material is a difficult task, but there are different methods to overcome the hurdles introduced by the problem of the size and the influence of handling substrate. A direct approach to move through the heat dissipation relies on the so-called optothermal Raman spectroscopy study, where the spectroscopic monitoring of the frequency shift of the Raman-active phonons is used as a sub-micrometer local probe of the temperature, with no pattern or device structure needed on the sample. The same laser used for this scattering spectroscopy is a source of local heating, which can affect the phonon band of the measured sample. In this respect, we recently analyzed BP nanosheets mechanically exfoliated on thick SiO_2_/Si substrates. The laser-induced temperature can be deduced by Raman spectroscopy to achieve a progressive layer ablation by control. The effect of irradiation on the local temperature relies on the interplay of two different heat diffusion channels: the thermal conductivity within the material and the interface thermal conductance, which strictly depends on the substrate in use.

A further scientific advance would be to analyze the heat dissipation in a metal-supported BP. The effect of a metal substrate can differ from the SiO_2_/Si system in terms of interface thermal conductance. This is an important piece of information from a practical point of view because the metal interface is indeed a key-constituent of every electronic and optoelectronic device based on BP. The aim of this paper is to focus on BP exfoliated on a gold substrate, which can be taken as a representative case of a BP-metal junction. The experiment to determine the thermal interaction between the BP and the Au substrate consists of focusing a laser on the flake and observing the Raman shift variation with increasing power (and hence temperature). Similar to other techniques, this measurement can be interpreted indirectly with the help of a finite-element simulation. The simulation uses the BP thermal conductivity *k*, measured in the previous literature [19]. The comparison between experiment and simulation is carried out by a method based on the reconstruction of a virtual Raman spectrum. The outcome of this comparison is the first estimation of the value of the interface thermal conductance *G* between BP and gold.

## 2. Materials and Methods

### 2.1. Materials

**Figure 1:**
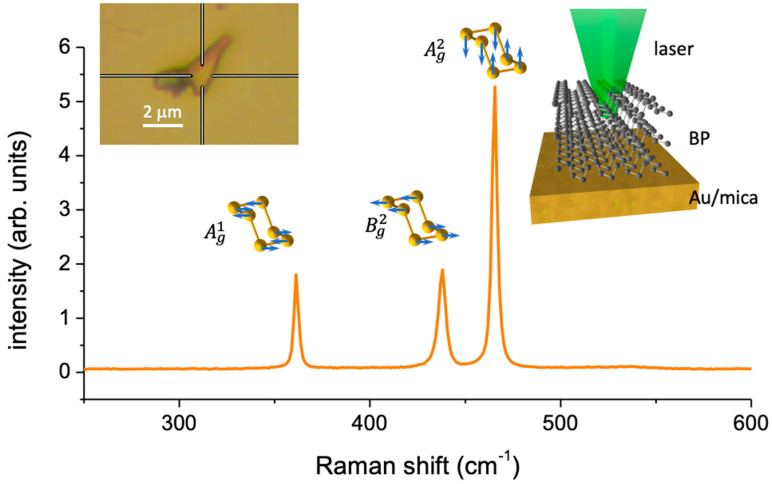
Raman spectrum of a black phosphorus nanosheet with the illustrations of the characteristic Raman modes. The insets show an optical image of a flake and the sketch of the measurement configuration.

We derived BP nanosheets from a commercially supplied BP crystal (HQ-Graphene, Groningen, The Netherlands) by means of tape exfoliation. The BP nanosheets are then transferred onto a commercially supplied 200 nm-thick (111)-terminated Au epilayer on a mica substrate (by Mateck GmbH, Juelich, Germany). The inset of Figure 1 shows one small flake on the substrate. Based on atomic-force microscopy, the thickness of the exfoliated flakes is measured to be in the range between 10 and 120 nm [20]. The thickness range explored in this paper is therefore always in the bulk range, where no effects due to quantum confinement take place. Fresh and aged samples did not show significant difference in the Raman spectrum. This is probably due to the fact that the presence of an oxide on the surface induces only a secondary effect in the dissipation of heat.

### 2.2. Raman Spectroscopy

We collected the Raman spectra from a T64000 spectrometer (Horiba Jobin-Yvon, Villeneuve-d’Ascq, France), a 640 mm focal length 2400 grooves/mm triple spectrometer operating in a single spectrometer configuration. The excitation laser was a 532 nm solid state laser with a maximum power of 60 mW on the sample. We used a 50× objective with a 0.75 numerical aperture because it showed a good trade-off between collection efficiency and stability in maintaining the focus for the whole measurement. We repeated the experiment on different flakes of the same family obtaining similar values in terms of spectral shift and band width.

### 2.3. Simulation

The finite element analysis was carried out by Matlab (The MathWorks Inc., Natick, MA, USA) based simulation code. The illumination process was modeled through a Gaussian lineshape with a radius determined by the numerical aperture of the objective and verified by scanning the edge of a sharp border of a metal-on-insulator sample. The simulation of the heating propagation was performed by the Partial Differential Equations Toolbox, solving a stationary heat equation in a cylindrical symmetry. The link between the experimental results and the simulation was elaborated by generating a virtual Raman spectrum and by considering every finite element of the simulation as a single scatterer with its own intensity, spectral position, and width [21,22].

## 3. Results and Discussion

### 3.1. Experimental Results of the Power Dependence of the Raman Spectra

A representative Raman spectrum of a BP nanosheet is reported in Figure 1, which shows the three characteristic Raman bands, termed $A_{g}^{1}$ (corresponding to the out-of-plane phonon mode), $B_{2g}$, and $A_{g}^{2}$ (corresponding to the phonon modes along the zigzag and armchair directions of the puckered BP edges).

**Figure 2:**
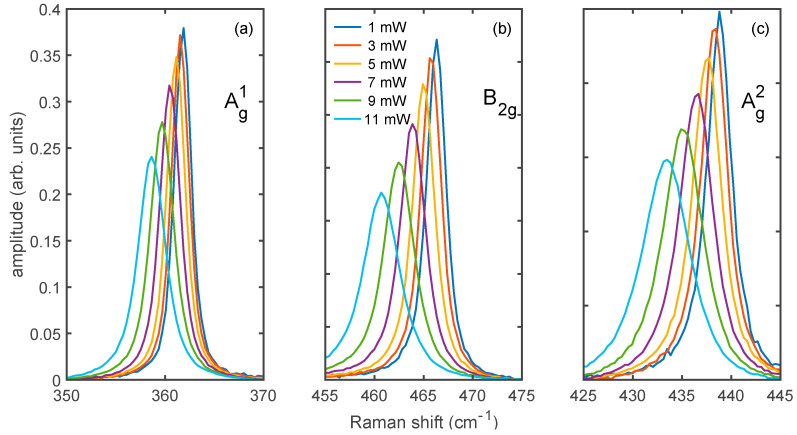
Normalized Raman bands $A_{g}^{1}$ (**a**), $B_{2g}$ (**b**), and $A_{g}^{2}$ (**c**) of a flake of black phosphorus on gold as a function of the increasing incident power.

Based on the BP nanosheets, our intent is to elucidate the heat dissipation by taking benefit from the laser irradiation as an excitation source for the Raman scattering and as a heat source for the local incrementation of the temperature. To this purpose, we focused the laser on the sample and gradually increased the released heat by adjusting the incident power while we simultaneously monitored the effect of the heating on the temperature and the structure of the sample by observing the changes in the Raman spectrum. No additional annealing was applied to the samples that were kept in room temperature conditions. The power-dependence of the Raman spectra is illustrated in Figure 2a–c, where the three main bands of BP, $A_{g}^{1}$, $B_{2g}$, and $A_{g}^{2}$, are normalized to their integrated intensity. We started with an incident power *p* as low as 1 mW. The power density associated with this value of *p* is low enough to not induce any change in the local temperature but high enough to collect a spectrum with a suitable signal-to-noise ratio. Lower values of *p* did not show any modification of the spectra, meaning that the induced heating was negligible as compared to room temperature. We repeated the experiment on the same spot with an increase in the *p* values by steps of 2 mW (under photodiode monitoring). The effect of the local heating in the sample can be readily observed from Figure 2 in the decreasing wavenumber shift Δω and increasing full width at half-maximum ΔГ with increasing power *p*. We stopped increasing the incident power when we observed a local thermal exfoliation of the phosphorus layer due to the layer ablation as previously reported in Reference [23]. The ablation onset can be detected as an abrupt decrease in the Raman band intensity, which is always accompanied by a simultaneous reduction in the absolute value of Δω and ΔГ. Here, consideration is paid to the pre-ablation regime, i.e., with power lower than a critical threshold over which BP layers undergo sublimation, in order to focus on the heat dissipation with no structural degradation occurring. This approach allowed us to sweep the power back and forth with no impact or modification on the BP.

**Figure 3:**
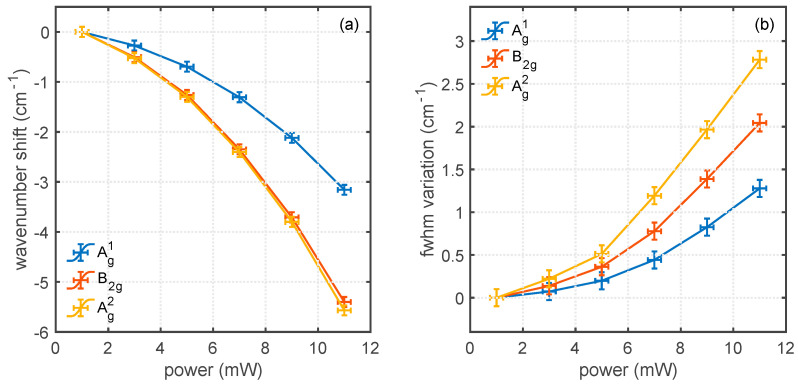
Modification of the three main Raman bands of black phosphorus on gold as a function of the illumination power. (**a**) Relative decrease in central wavenumber Δω. (**b**) Variation of the full width at half-maximum ΔΓ.

The fit to the spectra in Figure 2 with pseudo-Voigt functions is reported in Figure 3. Figure 3a shows that the decrease in Δω associated with heating is observed in all the spectra, although with a higher value for $A_{g}^{2}$ and $B_{2g}$ bands, with respect to the $A_{g}^{1}$ band. The similar behavior of the three bands in this experiment suggests that, although the same thermal information is carried by all the phonons, the $A_{g}^{2}$ mode appears to be the most sensitive to probe heat-related distortions of the phonon spectrum. The change in spectral position Δ*ω* is the sum of three contributions Δ*ω_A_*, Δ*ω_E_*, and Δ*ω_S_*, which are the shifts induced by anharmonic effects, thermal expansion, and strain due to lattice mismatch with the substrate, respectively. The most important is usually the anharmonic effect [24], whereas the thermal expansion and the strain contributions usually play a minor role. Figure 3b reports the increase in the linewidth. In this case, the three bands are also differently affected, and the most sensitive to heat is the $A_{g}^{2}$ band. Previously, we suggested that the increase in linewidth can be a better indicator of the local temperature since it is not affected by thermal expansion and interlayer stress.

Unlike the case of BP on SiO_2_ and Si, the curves in Figure 3a,b also show the presence of a non-linear component. This could be explained in principle by a variation of the thermal conductivity of phosphorus, gold, or their interface as a function of the increasing temperature. It is then interesting to elucidate what is the role of a metal substrate and its interface starting from the power dependence of the Raman spectral parameters. In this view, a better understanding of the experiment and a tentative extraction of the value of the interface thermal resistance must involve the simulation of the heating of the whole stack through a finite-element method.

### 3.2. Distribution of the Heat in the Sample and Simulation of the Raman Spectra

In order to gain further understanding of the thermal resistance at the interface, we performed a finite-element simulation of the heat distribution of the illuminated flake and substrate. The stack was simulated with a set of three layers of BP, gold, and mica with a cylindrical symmetry. For the BP layer, we used a 2.0 µm radius, whereas gold and mica were simulated by a 2.5 µm radius. We did not observe any significant variation by increasing these values further, and therefore from the point of view of the simulation the flake behaves as an infinitely large layer. The size of the flakes analyzed experimentally was indeed larger than this value. The thickness of the three considered layers were 30 nm, 200 nm, and 100 nm, respectively. The thickness of the BP layer was chosen to match the value of the flake reported in Figure 2 and Figure 3. The simulated thickness of the handling mica wafer is much lower than the true physical thickness, but this approximation is necessary to keep the calculations reasonably fast and it is justified by the fact that we did not observe any change after repeating the simulation with a 200 nm-thick mica layer. This suggests that the role played by the mica substrate can be disregarded with respect to the gold layer. As a boundary condition, we set the bottom temperature of the mica at the room temperature of 24 °C.

The thermal behavior of BP is complicated by its anisotropic character. The thick BP layer exhibits an in-plane body conductivity *k*_in-plane_ of 20 and 40 Wm^−1^ K^−1^ in the armchair and zigzag values of conductivity, respectively [17]. Since we were using a cylindrical symmetry model, we took the average conductivity value 〈*k*_in-plane_〉 = 30 Wm^−1^ K^−1^ as an input to the simulation. For the vertical transmission of out-of-plane thermal conductivity *k*_out-of-plane_ = 4 Wm^−1^ K^−1^ is taken from Ref. [25]. We introduced the effect of a finite interface thermal conductance *G* in the model by adding a fictitious infinitesimal layer between BP and gold. This layer (with a thickness *t*_G_ of 1 nm in the simulation) was characterized by a thermal conductivity *k*_G_ obtained by the product between the true interface thermal conductance *G* of the BP/gold interface and the thickness of the layer.

**Figure 4:**
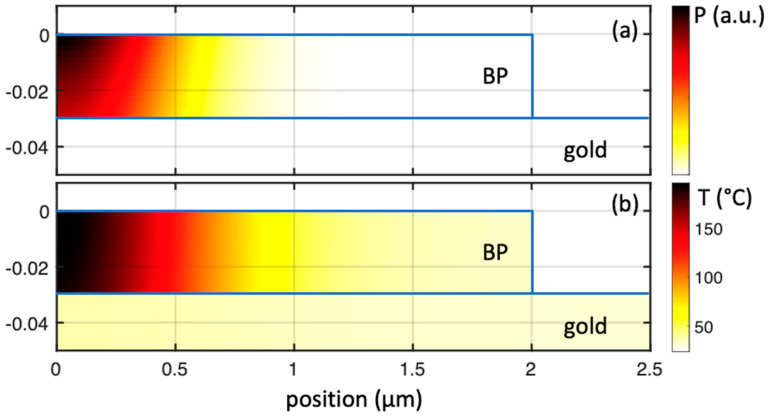
Finite-element simulation of the heating effects of a laser focused on a 30 nm thick layer of black phosphorus on top of a gold-on-mica substrate. The figure is magnified in the black phosphorus region. The position in (0, 0) corresponds to the center of the illumination. The vertical scale is expanded with respect to the horizontal scale for clarity. (**a**) Local illumination power density. (**b**) Spatial distribution of the induced local temperature.

For the comparison of the simulation with the experimental results, we used a method which is not based on the spatial average of the temperatures, but rather on the sum of all the spectra generated by the single *i*-th finite elements.

$$ℛ=\underset{i}{\sum }A_{i}ℒ\left(\omega_{0}+\Delta \omega_{i}\right)$$

where ℛ is the amplitude function of the wavenumber w representing the total simulated Raman spectrum. The BP sample reported in Figure 4 is divided evenly in elements of 2 × 2 nm^2^. Each of these infinitesimal elements is treated as an individual *i*-th scatterer producing an infinitesimal spectrum which is a Lorentzian curve ℒ centered in $\omega_{i}=\omega_{0}+\Delta \omega_{i}$ and with an amplitude $A_{i}$. The choice of a Lorentzian is due to the shape of the typical Raman scattering band.

The amplitude $A_{i}$ is determined by the local illumination and collection efficiency. For the illumination of the sample, we used a Gaussian distribution with a diffraction-limited radius. The choice of a Gaussian function for the illumination intensity is a generally accepted approximation of the shape of the focal field. The interference effects are neglected to a first approximation. The resolution was checked with the usual method of scanning on the edge of a sample with a sharp change of reflectivity. The probability of collection of the scattered material can be considered to be proportional to the illumination density since the difference between the excitation wavelength (532 nm) and the scattered wavelength (about 545 nm for the $A_{g}^{2}$ mode) is less than 3%. 

The spectral position of each Lorentzian ℒ is instead determined by the local temperature. The effect of the temperature is to shift the center of the Lorentzian distribution of a quantity Δω with respect to $\omega_{0}$, the room temperature value for the $A_{g}^{2}$ mode. The coefficient c linking Δω to the temperature for the $A_{g}^{2}$ mode can be found in the literature as c = −0.0263 cm^−1^ K^−1^ [26].

**Figure 5:**
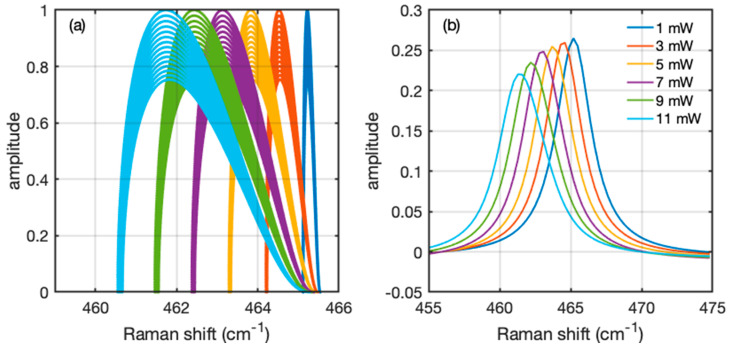
Simulation of the Raman spectrum from the distribution of temperatures. (**a**) Each point represents the wavenumber and the amplitude of the contribution of each finite element of the thermal simulation. Each color represents a different excitation power ranging from 1 to 11 mW, with the same color code as Figure 2. The distributions are normalized to each maximum amplitude. (**b**) Summation of all the infinitesimal spectra generated from the elements of (**a**), giving rise to spectra that can be compared to the experimental results. The spectra are normalized to their integrated intensity.

The sum of all the infinitesimal spectra is a spectrum ℛ which can be directly compared with the experimental data. Proceeding in this way improves the method of averaging the temperature, since it is not always obvious that the average temperature is the one that can be readily retrieved from the experimental spectrum. As an example of this procedure, we report in Figure 5 the simulation of a Raman spectrum of the sample represented in Figure 4 for the case of an illumination ranging from 1 to 11 mW. Figure 5a represents the contribution of each *i*-th element to the final spectrum. In this plot each *i*-th element is represented by a point characterized by a normalized amplitude $A_{i}$ and a Raman shift $\omega =\omega_{0}+\Delta \omega_{i}$. As such, the plot represents the distribution of the single contributions of each simulated element. The summation of all the individual contributions is reported in Figure 5b, which is the simulation to be directly compared with Figure 2. The shape and shift of the simulated spectrum is a function of the input parameters of the simulation. Since all the thermal parameters of the simulation are taken from the literature, the only parameter that can be modified is the interface thermal conductance *G*. As a result, the experimental behaviors in Figure 3 are reproduced when setting the value of *G* = 10^7^ Wm^−2^ K^−1^. Since the simulation involves the approximations mentioned before and the physical parameters used in the simulation taken from the literature are also associated with uncertainties, this value of *G* must be taken as a rough estimation of the interface thermal conductance of BP nanosheets on Au. It is possible to recognize in the spectra that higher values of illumination and heat lead to a shift of the positions towards lower wavenumber and a simultaneous slightly asymmetric deformation of the distribution.

## 4. Conclusions

In this work, we analyze the thermal behavior of BP on a metal substrate, and we make an estimation of the thermal interface conductance. The heat dissipation in Au-supported BP nanosheets is investigated by comparing laser power-dependent Raman spectra with a finite element simulation of the local heat diffusion induced by the power irradiation. We found that the power dependence of the Raman shift and width of the relevant Raman bands of the BP nanosheet, i.e., the $A_{g}^{1}$, $B_{2g}$, and $A_{g}^{2}$ modes, can be reproduced by an effective interface thermal conductance of G = 10^7^ Wm^−2^ K^−1^, resulting from the match between the experimental data and the simulation. This value suggests that interface effects should be considered when studying the heat diffusion in a BP system.

## Data Availability

Data can be available upon request from the authors.
